# Antimicrobial Glucose Chlorhexidine Dressings vs. Sterile Dressings in Preventing Catheter‐Related Infections During Continuous Renal Replacement Therapy: A Randomized Controlled Trial

**DOI:** 10.1111/sdi.70042

**Published:** 2026-07-31

**Authors:** Xuemei Zhang, Fang Wang, Yi Zheng, Xiankun Sun, Li Lin, Xue Tang, Zhiwen Chen, Ling Zhang

**Affiliations:** ^1^ Department of Nephrology West China Hospital, Sichuan University/West China School of Nursing, Sichuan University Chengdu Sichuan China; ^2^ Department of Nephrology West China Hospital, Sichuan University Chengdu Sichuan China

**Keywords:** catheter‐related bloodstream infections, continuous renal replacement therapy, glucose‐chlorhexidine antimicrobial dressings

## Abstract

**Background:**

Catheter‐related bloodstream infections (CRBSIs) are a major cause of complications in critically ill patients receiving continuous renal replacement therapy (CRRT), especially those with nontunneled catheters. These infections can lead to longer hospital stays, higher costs, and worse patient outcomes. Although sterile dressings are commonly used to prevent CRBSIs, antimicrobial dressings, such as those containing chlorhexidine, may offer better protection. This study aimed to compare the effectiveness of antimicrobial glucose chlorhexidine dressings and sterile dressings in preventing CRBSIs in patients receiving CRRT.

**Methods:**

A prospective, randomized controlled, single‐blind study was conducted. All participants were kept blinded to group assignment. We enrolled patients admitted to the Intensive Care Unit (ICU) of West China Hospital, Sichuan University between September 2022 and November 2023 who were scheduled for vascular catheter placement before receiving continuous renal replacement therapy (CRRT). The participants were randomly allocated to either the intervention group (receiving glucose chlorhexidine antimicrobial dressing) or the control group (receiving sterile dressing) via a random number table. The dressing was changed every 7 days for the glucose chlorhexidine antimicrobial group and every 48 h for the sterile dressing group, in accordance with the guideline. Dressings were replaced immediately if they became rolled, loose, or visibly soiled. The primary outcomes were the local infection rate at the catheter insertion site, suspected catheter‐related bloodstream infections (CRBSIs), incidence of CRBSIs, length of hospital stay, ICU stay duration, and hospital mortality rates.

**Results:**

A total of 520 patients were screened, and 410 patients were ultimately included in the study, with 205 patients in each group (glucose chlorhexidine antimicrobial dressing group and sterile dressing group). The incidence of local infection at the catheter insertion site was 40.0% in the sterile dressing group, which was significantly greater than the 15.6% reported in the glucose chlorhexidine antimicrobial dressing group (*p* < 0.001). The total CRBSI rate in the intervention group was significantly lower (6.3%) than that in the control group (13.7%) (*p* = 0.014). No significant differences were found between the two groups in terms of suspected catheter‐related bloodstream infections (CRBSIs), CRBSI incidence, length of hospital stay, ICU stay duration, or hospital mortality rates (*p* > 0.05). The Cox proportional hazards model suggested that patients in the control group were at a substantially greater risk of local infections and CRBSIs over time than those in the intervention group.

**Conclusion:**

Glucose chlorhexidine antimicrobial dressings significantly reduce catheter insertion site infections and overall CRBSI incidence, demonstrating their clinical value in preventing infection in patients receiving CRRT.

## 1 Introduction

Continuous renal replacement therapy (CRRT) is a widely utilized blood purification technique in critically ill patients, particularly those with acute renal failure, sepsis, acute respiratory distress syndrome, and other life‐threatening conditions. It plays a crucial role in maintaining fluid and electrolyte balance while removing metabolic waste products [1], making it a vital life‐support treatment for critically ill individuals [2]. The choice and management of vascular access are pivotal aspects of CRRT, directly impacting treatment outcomes and patient prognosis. Nontunnelled catheters without Dacron cuffs are commonly used for vascular access because of their ease of placement, safety, and favorable hemodynamic stability [3, 4]. However, despite their broad clinical application, these catheters are associated with several risks and complications, most notably, catheter‐related bloodstream infections (CRBSIs), which occur at incidence rates ranging from 3.8‰ to 6.6‰ [5]. CRBSIs can significantly deteriorate patient conditions and, in severe cases, may be life‐threatening.

To mitigate the risk of CRBSIs, an increasing number of studies have focused on optimizing the care and management of the catheter insertion site. Glucose chlorhexidine antimicrobial dressings, a novel antimicrobial material, have shown considerable efficacy in reducing the incidence of CRBSIs in various clinical settings. Research indicates that glucose chlorhexidine dressings continuously release chlorhexidine, forming an antimicrobial barrier that reduces bacterial attachment at the catheter insertion site, thereby effectively preventing infection [6, 7]. Compared with traditional sterile gauze dressings, glucose chlorhexidine antimicrobial dressings offer prolonged antimicrobial effects, reduce the frequency of dressing changes, and alleviate the nursing workload.

Therefore, this prospective randomized controlled study aims to compare the efficacy of glucose chlorhexidine antimicrobial dressings with traditional sterile dressings in nontunneled catheters without Dacron cuffs.

## 2 Methods

### 2.1 Study Design and Ethics

**FIGURE 1:**
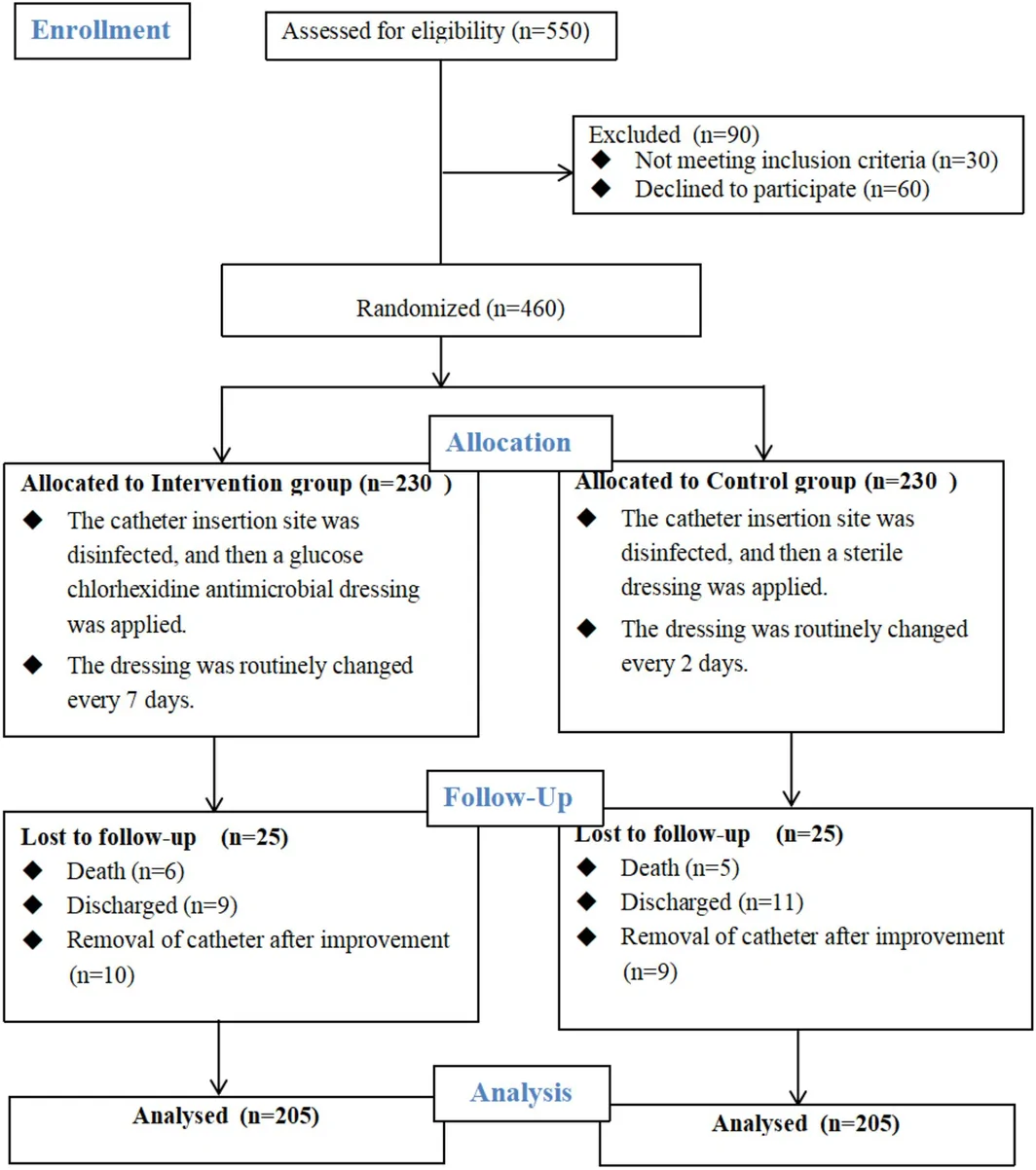
CONSORT 2010 flow diagram.

This prospective, randomized controlled, patient‐blinded study enrolled patients who received CRRT in the intensive care unit (ICU) of West China Hospital, Sichuan University between September 2022 and November 2023. Randomization was performed via SPSS 27.0 software to generate random numbers, which were placed in opaque, sealed envelopes labeled with serial numbers. The envelopes were opened sequentially by an independent research nurse not involved in the outcome assessment, who then prepared the corresponding dressing according to the allocation. Patients were assigned on the basis of the sequence of CRRT initiation at a 1:1 ratio to either the chlorhexidine gluconate antimicrobial dressing group or the sterile gauze group. Patients were unaware of their group assignment. The study flowchart is depicted in Figure 1.

The study protocol was approved by the Ethics Committee of West China Hospital, Sichuan University (Approval No. 2021‐1137), and it was registered with the Chinese Clinical Trial Registry (ChiCTR2400080678).

### 2.2 Patient Recruitment

Prior to patient allocation and the commencement of any study‐related procedures, informed consent was obtained from all participants or their legal guardians. Patients meeting the following criteria were enrolled between September 2022 and November 2023. The inclusion criteria were as follows:

1. Age between 18 and 70 years (18 ≤ age ≤ 70);
2. No history of allergy to chlorhexidine gluconate;
3. Intact skin at the catheter insertion site, with no trauma or signs of infection;
4. Willingness to participate and provision of written informed consent.

The exclusion criteria were as follows:

1. The use of steroids, immunosuppressants, or other medications within 30 days prior to the study:
2. Diagnosis of catheter‐related bloodstream infection (CRBSI) or any local infection:
3. Presence of skin lesions, such as rashes or drug eruptions, at the catheter insertion site, making it unsuitable for dressing application.

### 2.3 Procedures

A femoral venous catheter with a double‐lumen catheter (13 Fr, 250 mm; GDHK‐1325, Baxter International Inc., Deerfield, Illinois, USA) was inserted by a physician with clinical experience under ultrasound guidance. The prescribed dose of 25–35 mL/kg/h was followed. Nurses skilled in CRRT techniques administered CRRT to all participants.

#### 2.3.1 Intervention Group (Chlorhexidine Gluconate Antimicrobial Dressing Group)

The area surrounding the catheter insertion site was disinfected with 0.1% povidone‐iodine solution, covering a minimum area of 15 cm × 15 cm. After the disinfectant dried, a Chlorhexidine Gluconate Antimicrobial Dressing (TegadermTM CHG 1657R, 11 × 8.5 cm; 3 M, Neuss, Germany) was applied. Following the relevant guidelines [8, 9], the dressing was changed every 7 days or replaced immediately if it became rolled, detached, or visibly soiled.

#### 2.3.2 Control Group (Sterile Gauze Group)

The same disinfection procedure was followed, but a sterile gauze dressing (Medical Sterile Absorbent Cotton Pads, 11 × 9 cm; Chengdu Medical and Sanitary Materials Factory, China) was applied, which was replaced every 48 h or earlier if it became curled, detached, or visibly soiled [8, 9].

### 2.4 Sample Size

A pilot study revealed that the incidence of catheter‐related local infections in the sterile gauze group was 55%. Assuming that chlorhexidine gluconate antimicrobial dressing could reduce the incidence by 40%, the required sample size for each group was calculated via the two‐independent sample comparison formula, with a two‐tailed α = 0.05 and β = 0.2. The estimated sample size per group was 171 patients. Considering a 20% dropout rate, 205 patients were included in each group.

### 2.5 Data Collection

Clinical and demographic characteristics, including age, sex, weight, BMI, disease diagnosis, comorbidities, and catheter insertion site, were collected. In particular, laboratory test results obtained within 24 h of catheter insertion, including CD4 + T cells, white blood cell (WBC) count, C‐reactive protein (CRP), interleukin‐6 (IL‐6), procalcitonin (PCT), creatinine (Cr), and estimated glomerular filtration rate (eGFR), were also recorded. Additionally, the Sequential Organ Failure Assessment (SOFA) score, catheter indwelling time, and catheter locking solution usage were also documented.

### 2.6 Outcome Measures

#### 2.6.1 Local Infection at the Insertion Site

Defined by the presence of purulent discharge or erythema [10].

#### 2.6.2 Suspected Catheter‐Related Bloodstream Infection (CRBSI)

Clinical signs included fever, chills, and/or hypotension, with no obvious source of infection other than the catheter.

#### 2.6.3 Catheter‐Related Bloodstream Infection (CRBSI)

CRBSI is defined as a primary infection occurring during catheterization or within 48 h after catheter removal. Symptoms include fever (> 38°C), chills, hypotension, and a positive blood culture for bacteria or fungi or matching pathogens from the catheter tip and peripheral blood cultures with similar antibiotic susceptibility profiles [10, 11, 12].

#### 2.6.4 Hospitalization Time, ICU Stay, and Mortality

Length of hospital stay, length of ICU stay, and in‐hospital mortality events were recorded.

### 2.7 Statistical Analysis

The data were analyzed via SPSS 27.0 software. For normally distributed continuous data, the mean ± standard deviation (SD) was used, and group comparisons were performed via independent sample *t*‐tests. Non‐normally distributed data are expressed as the median and interquartile range (IQR), with group comparisons made via the Mann–Whitney U test. Categorical data are expressed as frequencies and percentages, and comparisons between groups were conducted via the chi‐square test or Fisher's exact test. A Cox proportional hazards regression model was used to analyze the association between catheter dwell time and the risk of catheter‐related infections.

A *p* value of < 0.05 was considered to indicate statistical significance.

## 3 Results

### 3.1 Baseline Characteristics of Patients

A total of 550 patients were screened for this study; 30 patients did not meet the inclusion criteria, and 60 patients declined to participate. Ultimately, 410 patients were enrolled, with 205 patients in the intervention group and 205 patients in the control group (Figure 1). There were no statistically significant differences between the two groups in terms of age, sex, BMI, comorbidities, or other baseline characteristics (*p* > 0.05), as shown in Table 1.

**TABLE 1:** Baseline characteristics of the patients enrolled in the study (*n* = 410).

Characteristic	Control Group (*n* = 205)	Intervention Group (*n* = 205)	Z/χ^2^/t	*p*
Age, [^2212^x ± s, year]	51.6 ± 14.3	51.7 ± 15.4	t = −0.026	0.979
Male, n(%)	156.0 (76.1)	160.0 (78.1)	χ^2^ = 0.221	0.638
BMI, ^2212^x ± s,kg/m^2^	25.0 ± 4.7	25.5 ± 4.9	t = 1.022	0.307
Comorbidities, *n*(%)				
Diabetes	36 (17.6)	47 (22.9)	χ^2^ = 1.828	0.176
Hypertension	59 (28.8)	58 (28.4)	χ^2^ = 0.006	0.938
Chronic kidney disease	83 (40.5)	79 (38.5)	χ^2^ = 0.163	0.686
Pancreatitis	49 (23.9)	54 (26.3)	χ^2^ = 0.324	0.569
Liver failure	17(8.3)	20(9.8)	χ^2^ = 0.267	0.605
Reasons for CRRT				
Chronic kidney disease	83 (40.5)	79 (38.5)	χ^2^ = 0.163	0.686
Acute kidney injury	104(50.7)	111(54.1)	χ^2^ = 0.479	0.489
Fluid volume overload	18(8.8)	15 (7.3)	χ^2^ = 0.297	0.586
Femoral venous catheter position, *n*(%)			χ^2^ = 0.040	0.837
Right femoral	130.0 (63.4)	132.0 (64.4)		
Left femoral	75.0 (36.6)	73.0 (35.6)		
Laboratory data at CRRT initiation				
CD4^+^T, [M (Q_1_, Q_3_)]	141.5 (70.8, 254.5)	97.5 (50.5, 260.0)	Z = −0.955	0.340
WBC,[M (Q_1_, Q_3_)]	10.6 (6.7, 15.6)	11.4 (6.9, 18.9)	Z = −1.048	0.295
CRP, [M (Q_1_, Q_3_)]	34.7 (17.9, 62.3)	31.2 (18.2, 67.6)	Z = −0.636	0.525
IL‐6, [M (Q_1_, Q_3_), pg./mL]	216.9 (70.7, 942.0)	178.1 (76.7, 910.0)	Z = −0.218	0.828
PCT, [M (Q_1_, Q_3_), ng/mL]	6.1 (1.7, 18.9)	4.5 (1.4, 16.2)	Z = −1.614	0.107
Cr, [M (Q_1_, Q_3_), μmol/L]	186.0 (108.0, 321.0)	184.0 (100.0, 301.0)	Z = −0.210	0.834
eGFR, [M (Q_1_, Q_3_), mL/min/1.73 m^2^]	34.8 (17.9, 62.3)	31.2 (18.2, 67.6)	Z = −0.132	0.895
SOFA Score, [M (Q_1_, Q_3_)]	12.0(6.0,15.0)	10.0 (7.0,14.0)	Z = −1.460	0.144
Catheter indwelling time, [M (Q_1_, Q_3_), day]	7.0 (3.5,1.0)	7.0 (4.0,10.0)	Z = −1.124	0.261
Catheter locking solution, *n*(%)			χ^2^ = 2.330	0.127
Citrate	180(87.8)	169(82.4)		
Heparin	25(12.2)	36(17.6)		

### 3.2 Comparison of Clinical Outcomes

The local infection rate at the catheter insertion site was 15.6% in the intervention group and 40% in the control group, indicating a statistically significant difference (*p* < 0.001) (Table 2). The total CRBSI rate in the intervention group was significantly lower (6.3%) than that in the control group (13.7%) (*p* = 0.014).

**TABLE 2:** Clinical outcomes in the study (*n* = 410).

Characteristic	Control group (*n* = 205)	Intervention group (*n* = 205)	Z/χ^2^	*p*
Local Infection at the Insertion Site, *n*(%)	82.0 (40.0)	32.0 (15.6)	χ^2^ = 30.380	< 0.001
Total CRBSI, *n*(%)	28(13.7)	13(6.3)	χ^2^ = 6.098	0.014
Suspected CRBSI, *n*(%)	19.0 (9.3)	10.0 (4.9)	χ^2^ = 3.010	0.083
CRBSI, *n*(%)	9.0(4.4)	3.0(1.5)	χ^2^ = 3.090	0.079
Gram‐positive bacterial infection	2.0(10.5)	0 (0)	χ^2^ = 1.131	0.288
Gram‐negative bacterial infection	7.0 (36.8)	3.0 (30.0)	χ^2^ = 0.136	0.713
Length of hospital stay, [M (Q_1_, Q_3_), day]	28.0 (17.0, 36.0)	26.0 (15.0, 42.0)	Z = −0.130	0.900
ICU stay, [M (Q_1_, Q_3_), day]	18.0 (9.0, 29.0)	17.0 (8.0, 31.0)	Z = −0.310	0.757
Hospital mortality, *n*(%)	136.0 (66.3)	127.0 (61.9)	χ^2^ = 0.860	0.354

No significant differences were detected between the two groups in terms of suspected CRBSIs, confirmed CRBSIs, hospital stay, ICU stay, or hospital mortality (*p* > 0.05) (Table 2).

### 3.3 Subgroup Analysis

The catheter insertion site had no significant effect on the incidence of local infection or CRBSI. Specifically, local infection occurred in 42 (28.4%) and 72 (27.5%) of patients in the left and right femoral vein groups, respectively (*p* = 0.846). Similarly, the CRBSI incidence was 11 (7.4%) in the left group and 18 (6.9%) in the right group (*p* = 0.831).

**FIGURE 2:**
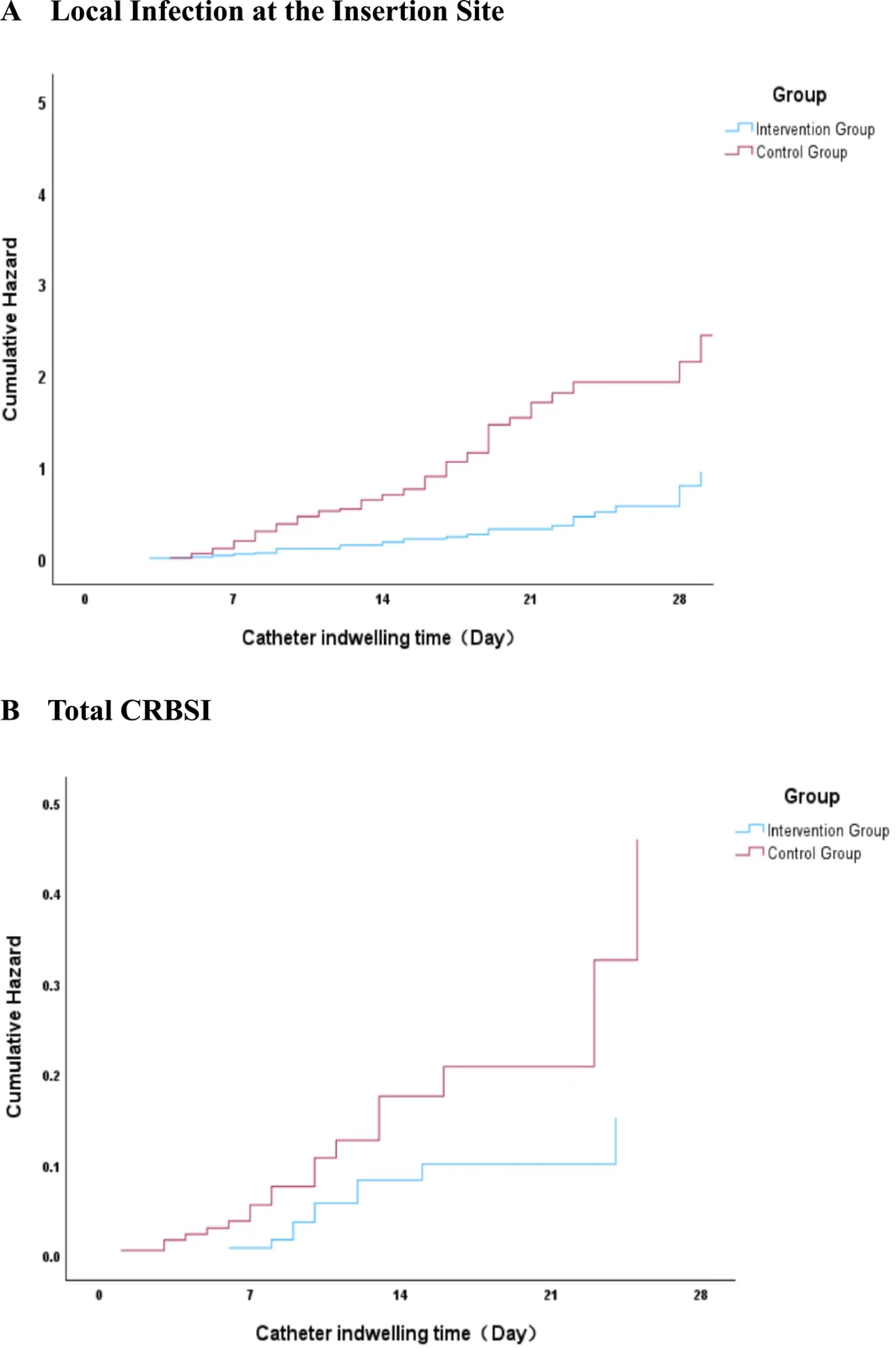
Cumulative risk of local infection at the insertion Site (A), Total catheter‐related bloodstream infection (B).

The catheter indwelling time was significantly longer in the intervention group (10.60 ± 8.50 days) than in the control group (8.60 ± 6.85 days), with the difference being statistically significant (*p* = 0.09). The cumulative hazard plot illustrates the differences in the risk of local infection at the catheter insertion site and total CRBSI between the two groups over time (Figure 2).

## 4 Discussion

This randomized controlled trial evaluated the efficacy of chlorhexidine gluconate antimicrobial dressing compared with sterile gauze in preventing catheter insertion site infections in patients undergoing CRRT. The results demonstrated that the intervention group, which received the chlorhexidine gluconate dressing, had a significantly lower incidence of local infections at the catheter insertion site (15.6%) than did the control group, which received sterile gauze (40.0%), with a *p* value < 0.001. Although there was a reduction in the total catheter‐related bloodstream infection (CRBSI) rate in the intervention group (6.3% vs. 13.7% in the control group, *p* = 0.014), no significant difference was observed in confirmed CRBSI cases between the two groups. Microbiological analysis of these confirmed CRBSI cases revealed a comparable distribution of causative organisms between the groups. Specifically, the proportions of infections caused by gram‐positive bacteria and gram‐negative bacteria were not significantly different (*p* > 0.05).

These findings suggest that chlorhexidine gluconate antimicrobial dressing is more effective in preventing local catheter site infections, likely because of its sustained release of antimicrobial agents that form a protective barrier against bacterial colonization [13, 14]. Chlorhexidine possesses broad‐spectrum antimicrobial properties and is capable of eradicating both gram‐positive and gram‐negative bacteria. Its slow‐release mechanism provides prolonged local antimicrobial effects, which are crucial for infection prevention.

Although the incidence of local infection at the insertion site was significantly lower in the Chlorhexidine Gluconate group than in the sterile gauze group, no significant difference was detected in the incidence of catheter‐related bloodstream infections (CRBSIs) between the two groups. This finding aligns with the study by Buetti et al. [15], which suggested that although chlorhexidine gluconate antimicrobial dressing effectively prevents local infections, its impact on CRBSI prevention may be limited. This discrepancy may stem from the multifactorial nature of CRBSIs, which involves a variety of factors, including catheter management, the patient's immune status, and the duration of catheter placement. Despite the greater frequency of dressing changes (every 48 h) in the sterile gauze group, it could not effectively prevent bacterial colonization and growth due to its lack of inherent antimicrobial properties compared with those of chlorhexidine gluconate. Additionally, gauze dressings are more prone to displacement or contamination, which could further undermine their clinical efficacy.

With respect to dressing change frequency, in this study, chlorhexidine gluconate antimicrobial dressing was replaced every 7 days, which significantly reduced the nursing workload and decreased the risk of infection. Similar studies have shown that reducing the frequency of dressing changes can effectively lower the risk of cross‐infections caused by frequent changes [16]. Moreover, the sustained antimicrobial effect of the chlorhexidine gluconate dressing reduces the likelihood of infection due to dressing displacement or contamination during patient care.

The predominant pathogens causing CRBSIs in this study were gram‐negative bacteria, a finding that is consistent with results from other relevant studies [17]. These findings suggest that the etiology of CRBSI is closely related to factors such as bacterial type and the duration of catheterization. Owing to their broad‐spectrum antimicrobial properties, chlorhexidine gluconate antimicrobial dressings offer effective protection against gram‐negative bacteria.

This trial exclusively used femoral access despite the KDIGO clinical practice guidelines (2012), which recommend the right internal jugular vein as the preferred access site [18], on the basis of the following considerations. First, multiple high‐quality studies and a meta‐analysis have shown no significant advantage of jugular vein catheterization over femoral site placement in critically ill patients requiring renal replacement therapy [19, 20]. Second, femoral vein catheterization is the predominant CRRT approach in most Chinese hospitals, where central venous catheters are generally placed in the internal jugular vein. Further studies incorporating both femoral and internal jugular vein catheterization are urgently needed to more comprehensively evaluate the efficacy of chlorhexidine gluconate dressings.

Although the study results revealed a lower incidence of CRBSI in the Chlorhexidine Gluconate group, the overall incidence of CRBSI was low in both groups, and no significant difference was detected between them. Similarly, no statistically significant differences were observed in key secondary clinical outcomes, including length of hospital stay and all‐cause mortality. This lack of discernible effects on these hard endpoints may be attributable to several interrelated factors. First, the trial was primarily powered to detect differences in infection rates and may be underpowered for outcomes such as mortality or hospital stay. Second, the multifactorial nature of mortality and prolonged hospitalization in critically ill patients—largely driven by underlying disease severity, organ dysfunction, and competing risks—likely dilutes the measurable impact of preventing a single complication such as CRBSI. Third, as noted, the sample size and baseline patient characteristics (such as age and immune function) may have influenced the observed outcomes. The median age of the patients in this study was between 50 and 60 years, which may limit the applicability of the results to younger populations. Age, as a potential confounding factor, could influence both CRBSI incidence [21] and overall clinical trajectory. Therefore, future large‐scale, multicenter studies that consider the effects of age and immune status, and are specifically designed to assess hard clinical endpoints may provide a more comprehensive understanding and further validate the clinical efficacy of chlorhexidine gluconate antimicrobial dressing.

## 5 Conclusions

In summary, this trial demonstrated that in critically ill patients undergoing CRRT, the use of chlorhexidine gluconate antimicrobial dressings significantly reduces the incidence of local catheter‐site infections compared with sterile gauze dressings. Although the study was not powered to detect differences in harder clinical endpoints such as mortality, the reduction in this key infectious complication underscores the intervention's value within a comprehensive catheter care bundle. Future research should focus on evaluating the cost‐effectiveness of this strategy and its impact on patient‐centered outcomes in larger, multicenter settings.

## Funding

This study is supported by the Sichuan Urban–Rural Integration Talent Development Research Foundation (HX‐H2209279).

## Ethics Statement

The study was approved by the Ethics Committee of West China Hospital, Sichuan University (Approval No. 2021‐1137), and it was registered with the Chinese Clinical Trial Registry (ChiCTR2400080678).

## Conflicts of Interest

The authors declare no conflicts of interest.

## Data Availability

All data are available upon reasonable request to the corresponding author.
